# The quest for vaccine-induced immune correlates of protection against tuberculosis

**DOI:** 10.18609/vac/2022.027

**Published:** 2022-07-29

**Authors:** Elisa Nemes, Andrew Fiore-Gartland, Cesar Boggiano, Margherita Coccia, Patricia D’Souza, Peter Gilbert, Ann Ginsberg, Ollivier Hyrien, Dominick Laddy, Karen Makar, M. Juliana McElrath, Lakshmi Ramachandra, Alexander C. Schmidt, Solmaz Shororbani, Justine Sunshine, Georgia Tomaras, Wen-Han Yu, Thomas J. Scriba, Nicole Frahm, BCG Correlates PIs Study Team, M72 Correlates PIs Study Team

**Affiliations:** 1 South African Tuberculosis Vaccine Initiative, Division of Immunology, Department of Pathology and Institute of Infectious Disease and Molecular Medicine, University of Cape Town, Cape Town, South Africa; 2 Vaccine and Infectious Disease Division, Fred Hutchinson Cancer Center, Seattle, WA, USA; 3 National Institute of Allergy and Infectious Diseases, National Institutes of Health; 4 GSK; 5 Bill & Melinda Gates Foundation, Seattle, WA, USA; 6 IAVI; 7 Bill & Melinda Gates Medical Research Institute, Cambridge, MA, USA; 8 Duke Human Vaccine Institute, Duke University, Durham, NC, USA

**Keywords:** tuberculosis, BCG, M72/AS01_E_, correlates of protection, systems vaccinology

## Abstract

Immunization strategies against tuberculosis (TB) that confer better protection than neonatal vaccination with the 101-year-old Bacille Calmette-Guerin (BCG) are urgently needed to control the epidemic, but clinical development is hampered by a lack of established immune correlates of protection (CoPs). Two phase 2b clinical trials offer the first opportunity to discover human CoPs against TB. Adolescent BCG re-vaccination showed partial protection against *Mycobacterium tuberculosis* (*Mtb)* infection, as measured by sustained IFNγ release assay (IGRA) conversion. Adult M72/AS01_E_ vaccination showed partial protection against pulmonary TB. We describe two collaborative research programs to discover CoPs against TB and ensure rigorous, streamlined use of available samples, involving international immunology experts in TB and state-of-the-art technologies, sponsors and funders. Hypotheses covering immune responses thought to be important in protection against TB have been defined and prioritized. A statistical framework to integrate the data analysis strategy was developed. Exploratory analyses will be performed to generate novel hypotheses.

## TB epidemiology

Tuberculosis disease (TB) is caused by *Mycobacterium tuberculosis* (*Mtb*), a mycobacterium transmitted by aerosol, which predominantly affects the lungs but can spread to any other organ [1]. Phylogenetic studies suggest that *Mtb* and humans co-evolved, possibly for the last 70,000 years [2]. For instance, TB may have killed up to 20% of the European and North American populations between the 17^th^ and 19^th^ century and was still responsible for the most annual deaths from a single pathogen globally until 2019 [3]. In 2020, global TB notification rates dropped to 5.8 million cases due to massive disruptions in testing and reporting caused by the COVID-19 pandemic, while the number of deaths increased, compared to 2019, to a total of 1.5 million [4].

Exposure to *Mtb* does not necessarily result in an established infection, but it is estimated that one-in-four humans are, or have been, infected with *Mtb* [5]. The population with viable *Mtb* infection is difficult to estimate, since no diagnostic test can directly detect *Mtb* in healthy individuals. *Mtb* infection status is inferred from the measurement of immune sensitization to *Mtb* antigens by tuberculin skin tests (TST) or, more recently, IFNγ release assays (IGRA) [6]. Recent epidemiological models suggest that once *Mtb* infection is established, a large proportion of individuals may clear or effectively control infection, possibly associated with TST and/or IGRA reversion to negative, while a smaller proportion may progress to TB disease over their lifetime [7]. Progression to disease is thought to typically occur relatively rapidly, within two years of primary infection; late progression may be associated with re-infection or weakening of the immune response several years after primary infection.

Novel and improved diagnostics, treatments and vaccines are urgently needed to address and ultimately end the TB epidemic [4]. Advocacy, political and economic commitments are also essential to address the common distorted perception that TB is an issue of the past, or that a deadly disease affecting mostly the poor is not a global emergency.

## TB vaccines

Bacille Calmette-Guerin (BCG) is the only available vaccine against TB [3]. This 101-year-old live, attenuated vaccine is routinely administered at birth in most countries [8], because it affords >80% protection against severe and disseminated TB disease, which has high mortality rates in children below 2 years of age [9]. Newborn BCG vaccination also confers partial protection against *Mtb* infection and pulmonary TB disease, but efficacy estimates vary greatly depending on age, geographical location, and previous sensitization to mycobacteria [9, 10]. BCG saves lives, especially when given in early life, via both pathogen-specific and pathogen-agnostic immunity that is under intense mechanistic investigation [11, 12], but has not stopped the TB epidemic; therefore, novel vaccines or immunization strategies are urgently needed.

While more efficacious and safer newborn BCG replacement vaccines are being tested to protect this vulnerable population, epidemiological modelling suggests that prevention of TB disease (POD) vaccines in adolescents and adults would have greater impact on TB transmission, and TB control in the general population [13]. Although the predicted impact of prevention of *Mtb* infection (POI) vaccines is lower compared to POD [14], measuring POI (or prevention of sustained infection, POSI) is also considered an innovative clinical trial end-point to efficiently provide proof-of-concept, since *Mtb* infection occurs much more often than TB disease [15]. However, PO(S)I vaccine efficacy may not necessarily predict POD, since immune responses required to prevent establishment of *Mtb* infection might be different from those required to prevent TB disease. Furthermore, partial prevention of infection may not translate to prevention of disease, since only a small minority of those who become infected typically progress to disease, and disease may preferentially occur in the subpopulation in whom infection occurred despite vaccination [1].

Rational design and clinical development of new TB vaccines have been hampered by a lack of immune correlates of protection (CoPs) [16]. In 2018, two phase 2 randomized placebo-controlled clinical trials reported partial efficacy of novel TB vaccination strategies, which, for the first time, provide the opportunity to discover CoPs against established *Mtb* infection and TB disease.

In the first trial, which aimed to assess POI, BCG re-vaccination of IGRA-negative adolescents provided 45.4% (95% CI, 6.4 to 68.1) protection against sustained IGRA conversion, defined as conversion to a positive test without reversion to negative status 3- and 6-months post-conversion [17], which is suggestive of established *Mtb* infection. Immunogenicity analyses showed significant boosting of BCG-specific Th1 and Th22 cells, as well as modest induction of NK responses after re-vaccination [18]. Antibody responses to BCG were not measured in this trial.

In the second, a POD trial, vaccination of IGRA positive adults with the investigational M72/AS01_E_ vaccine provided 49.7% (95% CI, 2.1 to 74.2) protection against microbiologically-confirmed pulmonary TB disease [19, 20]. Vaccination with M72/AS01_E_ induced robust M72-specific IgG and Th1 cellular responses [20], as well as NK cell responses [21].

Key features of these trials that affect the design and potential outcomes of the CoPs analysis are summarized in Table 1.

## TB immune correlates program

The TB Immune Correlates Program was initiated in 2018 for BCG-induced CoPs and in 2020 for M72/AS01_E_-induced CoPs. The Program provides the strategy and governance structure to enable discovery of CoPs from established *Mtb* infection (inferred from sustained IGRA conversion in the BCG re-vaccination trial) and TB disease (microbiologically confirmed in the M72/AS01_E_ trial) through a highly collaborative approach. The Program aims to: 1) Define hypotheses, biomarkers, and assays to be employed for the discovery of CoPs; 2) Develop the statistical framework and integrated data analysis strategy to evaluate the pre-specified hypotheses; 3) Pursue additional hypothesis-generating exploratory efforts; and 4) Ensure rigorous, efficient and streamlined use of the precious samples stored from these trials.

We hypothesize that vaccination induced a variety of immune responses comprising multiple immune cell subsets and, effector mechanisms, which synergistically contributed to the control of *Mtb* growth following infection, resulting in reduced rates of sustained IGRA conversion (or increased rates of IGRA reversion) in the BCG trial, or reduced rates of TB disease (M72/AS01_E_ trial) in vaccine compared to placebo recipients. Within this expectation, we aim to test a parsimonious set of pre-defined immunological hypotheses that are informed by the published literature, while allowing generation of additional hypotheses across a broad set of immunological compartments and mechanisms in a manner that rigorously controls the chance of false discovery. While the differences in trial designs and outcomes (Table 1) justify distinct hypotheses and experimental approaches, the overall alignment between the programs may enable identification of commonalities between the CoPs for POD and POSI.

Common between the two trials is the severe limitation of available samples (Table 1), particularly for cellular assays [peripheral blood mononuclear cells (PBMC)]. To ensure feasibility and robustness, and to reduce the number of outcomes tested, it was deemed necessary to apply a staged approach, whereby pilot studies are conducted on a limited set of samples [excluding samples from participants who met the respective clinical endpoint (i.e., cases)] to down-select hypotheses and assays for the primary analysis comparing endpoint cases and non-cases. To preserve statistical power in the primary analyses, results from the pilot study will be used to select assays that exhibit the characteristics required of a CoPs biomarker (see below). Hypotheses will be pre-specified and prioritized in the primary analysis. Exploratory analyses will follow to generate novel hypotheses for validation in ongoing or planned larger trials of BCG re-vaccination and M72/AS01_E_.

There are several key stakeholders of the TB Immune Correlates Program. Open calls-for-ideas were issued by the **Leadership Team**, separately for the BCG Program and the M72 Program. For both Programs, proposals from a large number of international investigators were evaluated by the **Scientific Advisory Committee**, and recommendations were made to the **Funders**, who further prioritized the most promising approaches to manage available resources. Established **Biospecimen Governance Committees** (including clinical trial sponsors and clinical site representatives) reviewed and approved sample access for the selected assays to the **Principal Investigators (PIs)** included in the **BCG and M72 Correlates Study Groups**. Results generated from the pilot studies will be evaluated and prioritized with a harmonized statistical approach, with analysis conducted by an independent statistical team [the Vaccines and Immunology Statistical Center (VISC) at Fred Hutchinson Cancer Center; Fiore-Gartland & Gilbert].

## Hypotheses and experimental approach

**Figure 1:**
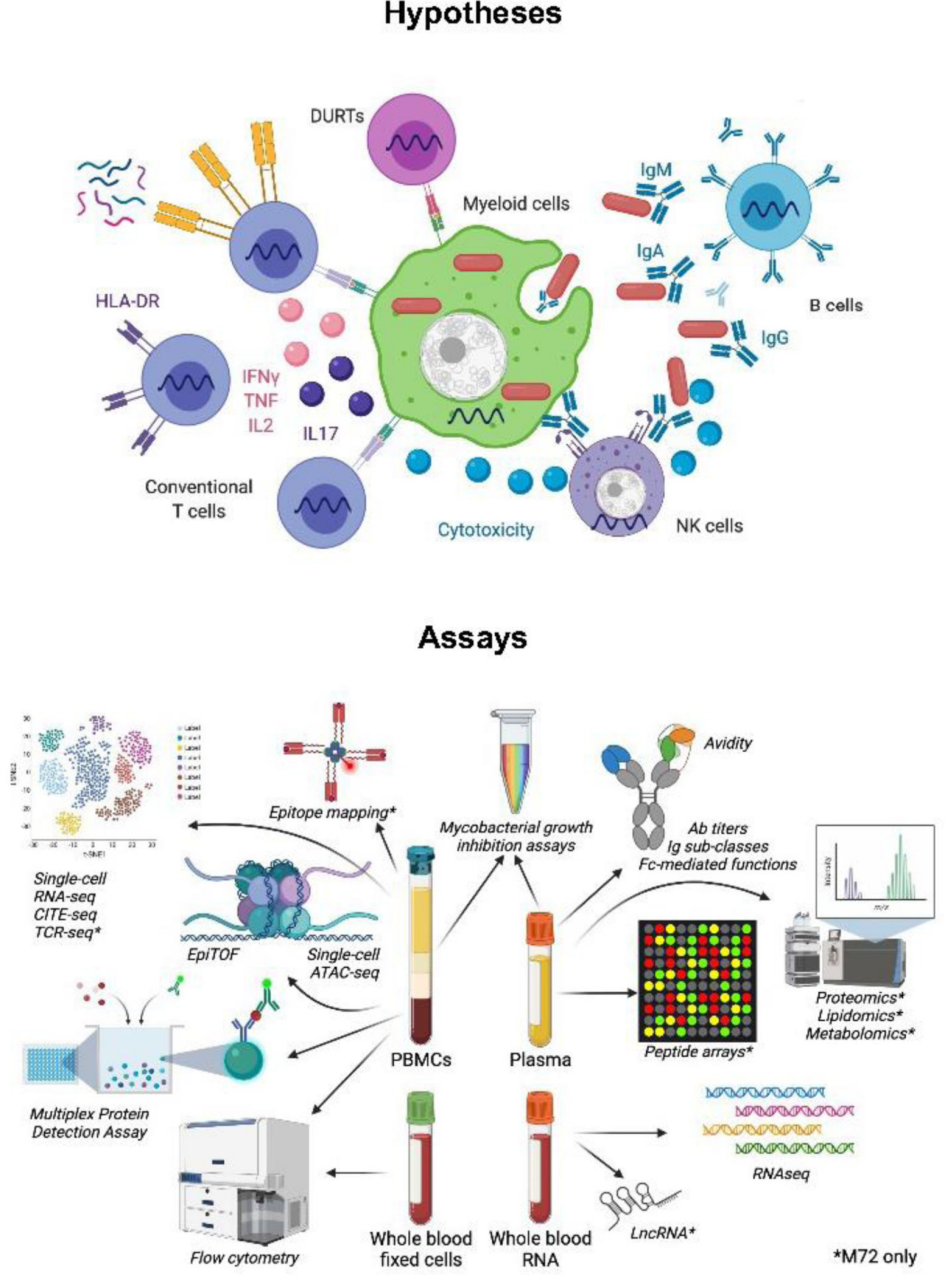
Hypotheses and experimental approach. Immune responses thought to be important for protection against TB have been identified and prioritized based on current knowledge. A systems immunology approach including several state-of-the-art assays will be employed to measure pre-selected outcomes as well as to generate new hypotheses (see text and Table 2 for more details). Most assays will be performed for both the BCG and M72/AS01_E_ programs, those performed only for the M72/AS01_E_ programs are denoted by *.

Immune responses to *Mtb* are complex, include many immune cell subsets and effector mechanisms of the immune system and are affected by the extra-cellular milieu and pre-existing immunity [22, 23]. Available evidence from pre-clinical models, particularly non-human primates (NHP), as well as cohort studies investigating immune correlates of risk for TB were considered to delineate the “immunological space” to be covered by the pilot studies and to outline the primary hypotheses to be tested using several state-of-the-art technological approaches (Figure 1 and Table 2). Primary hypotheses and outcomes will be further refined based on results from the pilot studies.

CD4 T cells are considered the cornerstone of immunity against *Mtb*. Their antigen-specificity, functional and phenotypic profiles, differentiation and activation status, as well the capacity to home to the lung parenchyma are all considered key features of successful anti-*Mtb* immune responses. Two independent NHP studies using alternative BCG vaccination routes, mucosal or intra-venous, recently showed protection against *Mtb* infection and TB disease [24, 25]. In both studies, increased abundance of mycobacteria-specific Th1/Th17 cells in the lung was associated with protection. No CoPs were identified in peripheral blood in the mucosal BCG study [24], while further experiments and analyses are ongoing in the intra-venous NHP model [26]. Even more recently, another NHP study showed that frequencies of Th1/Th17 and T1/T17 and cytotoxic T cells measured within individual granulomas are associated with differential control of *Mtb* infection [27].

These data support the primary hypothesis that mycobacteria-specific CD4 T cells displaying a hybrid Th1/Th17 phenotype are the main mediators of a protective immune response to *Mtb*.

Antigen-specific T cell responses will be measured primarily by intra-cellular cytokine staining and flow cytometry after antigen stimulation of PBMC from both trials [28] (McElrath & Andersen-Nissen).

For the M72 Program, additional approaches include sorting of M72-specific T cells and single-cell analyses including DNA-tagged antibodies, TCR sequencing and RNA sequencing using different platforms (Musvosvi, Scriba & Shalek, McElrath & Andersen-Nissen). Since M72/AS01_E_ only contains two *Mtb* antigens, further epitope mapping will be performed (Ernst & Altin), as well as broader measurements of immunomodulatory factors secreted upon PBMC stimulation with M72 by multiplex protein detection assay (McElrath & Andersen-Nissen).

Pathogen-specific antibodies are the primary CoPs for many effective vaccines, and multiple antibody functions beyond neutralization have been implicated in protection [29]. Studies conducted over a century ago showed some benefit of transferring serum from immunized horses to patients with TB, but antibodies to *Mtb* have not been consistently associated with protection (reviewed in [30, 31]). Recent studies using modern techniques have reinvigorated the hypothesis that antibodies may play a role in mycobacterial control, predominantly through Fc-receptor (FcR)-mediated functionality that can lead to killing of *Mtb* in infected cells [32]. The postulated beneficial role of NK cells reviewed in [33]) may well be linked to FcR-mediated effector functions.

These data support the co-primary hypothesis that antibody-dependent NK-cell activation contributes to control of *Mtb*.

Antibody sub-classes may also be important for protection, presumably via a different mechanism to antibody-dependent NK-cell activation. An NHP study that assessed protection against *Mtb* following mucosal BCG vaccination suggested that mycobacteria-specific IgA responses in bronchoalveolar lavage may correlate with protection [24]. In a different study, robust IgM responses induced in the blood and lungs by intravenous BCG vaccination of NHP were associated with protection and reduced mycobacterial burden [26].

Antibodies will be profiled for both Programs using several approaches, which include measurement of titers, sub-classes and avidity (Tomaras), as well as identification of Fc-mediated functions (Tomaras, Alter) and antibody-mediated mycobacterial growth inhibition (Alter). Peptides recognized by antibodies will be identified by phage immunoprecipitation (BCG, Rajan & Javid), peptide array (M72, Tomaras) and *Mtb* proteome microarrays (M72, Yee).

“Training” of monocytes/macrophages, and possibly NK cells, following innate immune activation by BCG or other pathogenic products has been well characterized [34] and has been associated with broad pathogen-agnostic protection against unrelated infections in humans [35]. AS01, the adjuvant used in the M72 vaccine, is known to potently activate the immune system [36]. It must, however, be acknowledged that trained immunity, as currently understood, may not be sufficiently long-lived to explain the durability of protection, which was observed up to 2 years after BCG re-vaccination and up to 3 years after M72/AS01_E_ vaccination.

Nevertheless, investigation of the role of innate – including trained – immunity is warranted considering its possible role in inducing and supporting the development of long-lasting adaptive immune responses.

Since epigenetic re-programming is a key feature underlying trained immunity, it will be measured by EpiTOF [37] (Utz & Khatri) and single-cell ATAC-seq (BCG: Barreiro; M72: Pulendran). Immune-gene priming long non-coding RNAs regulate the deposition of H3K4me3 at the promoters of immune genes [38] and will be measured by qPCR (M72, Mhlanga & Netea). Functional responses to heterologous stimuli will be measured by O-Link in the supernatant of stimulated PBMC in both trials (Utz & Khatri).

Technological advances have also contributed to a much more refined understanding of non-classical, so-called donor-unrestricted T (DURT) cells, which recognize non-protein-based antigens, and their role in mycobacterial control [39]. It was recently shown that the frequencies of DURT cells were not modulated by primary vaccination or re-vaccination with BCG [40]; however, the effects of BCG re-vaccination on their functional or phenotypic attributes have not yet been fully explored. Presentation not only of peptides but also non-protein-based antigens by the non-polymorphic antigen-presenting molecules CD1, MR1 and HLA-E may contribute to the pool of protective mycobacterial T-cell responses induced by BCG re-vaccination. While clear correlations with *Mtb* infection have not been described for these T cell subsets, enrichment of mucosa-associated invariant T (MAIT) cells has been observed in exposed individuals who remain uninfected [41], supporting the hypothesis that BCG-induced DURTs contribute to the early control of Mtb infection.

DURT cell frequencies and absolute counts will be measured in fixed whole blood by flow cytometry (Nemes & Scriba) and their function by intra-cellular cytokine staining after PBMC stimulation (McElrath & Andersen-Nissen).

In addition to generating outcome measures related to our primary biological hypotheses, several assays that provide an unbiased view of vaccine-induced changes and are thus likely to generate new hypotheses, or define the systemic milieu in which immune responses are induced by vaccination, will be employed.

A mycobacteria growth inhibition assay [42, 43] will be used (Joosten & Ottenhoff) as a functional readout to determine whether M72/AS01_E_ vaccination enhances overall mycobacterial growth control or even killing *in vitro*, as well as the relative contribution of cell-mediated and antibody-mediated immunity on this outcome.

Another hypothesis is that RNA sequencing (RNA-seq) analysis of whole blood, as well as single cells, will identify novel gene expression profiles, cellular subsets or pathways associated with protection against infection and/or disease (BCG: Shalek, M72: Musvosvi, Scriba & Shalek, McElrath & Andersen-Nissen). The whole blood RNA-seq dataset from the M72/AS01_E_ trial will also be mined to test the hypotheses that blood transcriptomic signatures of risk of TB, which allow identification of individuals in early stages of disease progression, or with subclinical disease [44–46], are elevated in M72 trial participants who developed TB within the first year [46] compared to non-cases (Scriba & Fiore-Gartland). Further, blood transcriptomic signatures associated with protection identified in animal models [47, 48] will be assessed for increased expression in non-cases compared to cases (Duffy & Nemeth).

To test the hypothesis that vaccination with M72/AS01_E_ elicits a multimolecular biosignature in participant plasma correlating with protection from active TB [49–51], lipidomics, metabolomics and proteomics approaches (Tafesse & Lewinsohn) as well as targeted measurements of apolipoproteins and complement proteins (Steen & Levy) will be undertaken.

Finally, phenotyping of whole blood leucocyte populations provides a snapshot of immune status in the absence of stimulation. Baseline expression of the activation marker HLA-DR on T cells [52], increased myeloid to lymphoid cell ratio [53] and reduced abundance of NK cells [54] have all been associated with increased risk of TB and will be assessed as biomarkers of risk of TB, or as CoPs. Whole blood immunophenotyping and absolute counts will be performed by flow cytometry [55] (Nemes and Scriba).

## Statistical considerations

The primary objective of the statistical analyses for the Pilot Study is to describe and rank assay readouts based on their potential to predict sustained infection or TB disease in future case-cohort analyses. Participants were selected for the Pilot Study at random from among the vaccine and placebo recipients that did not meet any of the trial endpoint criteria. Each lab will analyze a baseline and post-immunization (BCG: Day 70 post-BCG; M72/AS01_E_: Day 37, one week post-2nd injection) sample from a subset of vaccine (n=64) and placebo (n=22) recipients.

A head-to-head analysis will be conducted by the statistical team to quantify vaccine-induced immune responses, characterize performances of the assays, and identify a set of low-dimensional biomarkers. These analyses will inform decisions about which assays to prioritize for the Primary analysis of cases and non-cases.

Assay and readout performance will be evaluated using the following criteria:

1. Broad, biologically-relevant dynamic range among vaccine recipients after vaccination
2. Broad, biologically-relevant dynamic range among all participants at baseline
3. Low intra-individual temporal variability among placebo recipients
4. Large shift in the distribution among vaccine vs. placebo recipients (i.e., evidence of a vaccine-induced response)
5. Low technical measurement error
6. Low covariation among readouts within each assay, with a low-dimensional representation of the measured response
7. Low covariation of readouts across assays, reducing overlap and redundancy in immunological space
8. Broad coverage of relevant immune functions

**Figure 2:**
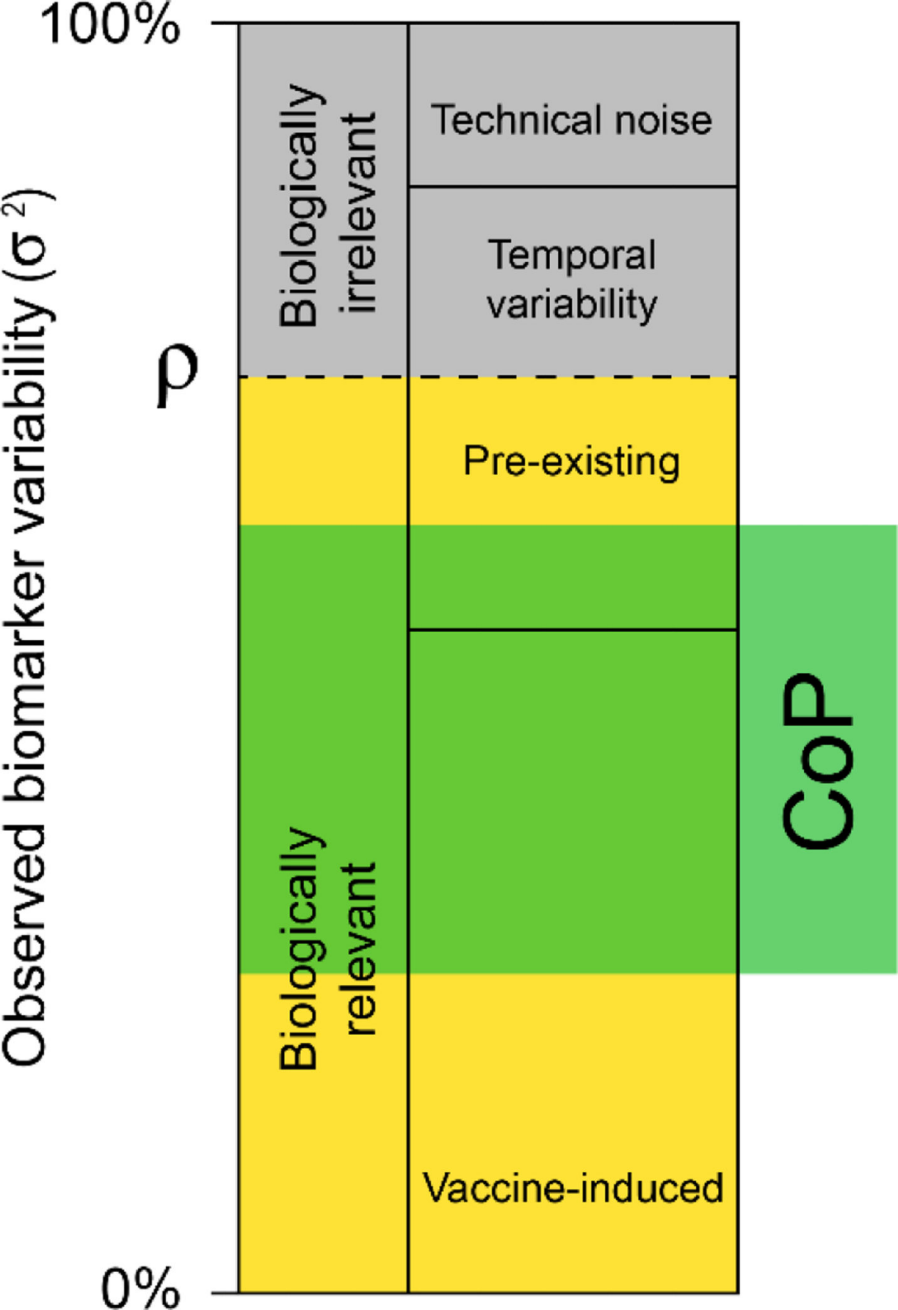
Partitioning of biomarker variability. The inter-vaccinee variance of each biomarker is made up of biologically relevant and irrelevant components. Irrelevant components contain measurement error and types of temporal variability that cannot be correlated with protection; measurement error can be estimated from technical replicates while temporal variability can be estimated from longitudinal sampling of placebos. Pre-existing and vaccine-induced variability in the marker can both be correlated with protection. The biologically relevant proportion of variation (ρ) can be affected by pre-existing factors like host-genetics (e.g., HLA, TLR SNPs), microbiome, or pre-existing immunity to *Mtb*.

With these criteria we will attempt to deconstruct the variance of each readout into the components of vaccine-induced and non-vaccine-induced variation. We will also seek to select assays and readouts that maximize the proportion of variability that could possibly correlate with TB risk or vaccine protection (Figure 2).

The sample size required to evaluate these outcomes in the Pilot studies, considering the expected variability of baseline BCG- and M72-specific responses as well as the number of cases available for the final analysis of each trial (Table 1), was determined to be 24 BCG and 12 placebo recipients for the BCG Program, and 40 M72/AS01_E_ and 10 placebo recipients for the M72 Program [56].

The goal of the Primary statistical analysis will be to evaluate each biomarker and combinations of biomarkers as correlates of risk (CoRs) and CoPs using a “case-cohort” design, including cases and non-cases from the randomized vaccine and placebo treatment groups.

**Figure 3:**
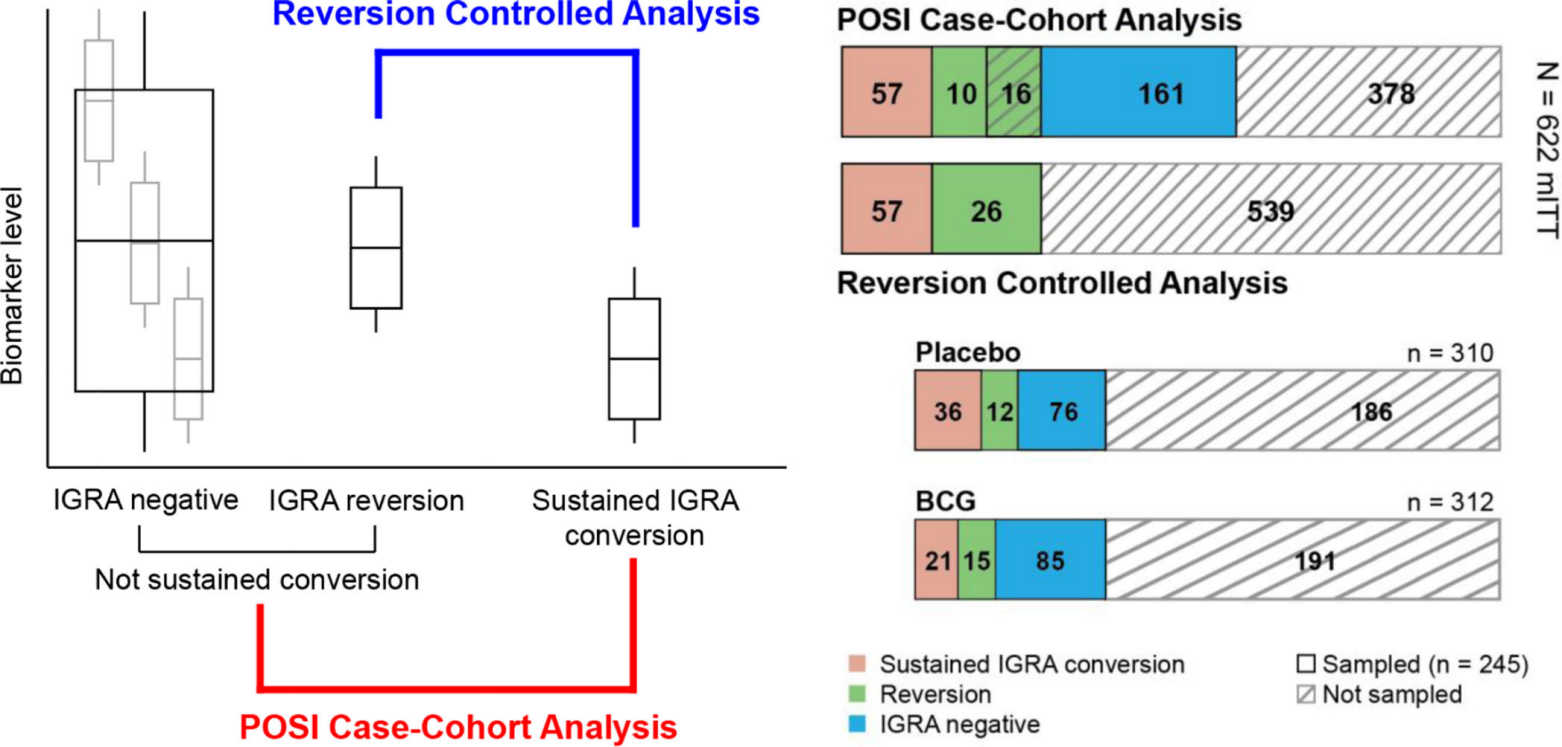
Sampling strategy for the BCG re-vaccination prevention of sustained infection (POSI) case-cohort immune correlates study. The correlates analysis will include two distinct comparisons: (1) Sustained IGRA conversion vs. no sustained conversion (POSI Case-Cohort Analysis), and (2) Sustained IGRA conversion vs. IGRA reversion (Reversion Controlled Analysis). The POSI Case-Cohort Analysis samples controls from participants without a sustained IGRA conversion, including a mixture of potentially exposed, unexposed and IGRA converted-reverted. In contrast, the Reversion Controlled Analysis conditions on initial conversion, excluding unexposed individuals and focusing on the phenotypic differences that distinguish reversion from sustained infection.

For the BCG Program, two different case-cohort analyses will be performed (Figure 3). In the “modified intent-to-treat (mITT)-controlled analysis” sustained IGRA converters will be compared to participants who did not display sustained IGRA conversion, i.e., a combination of participants who remained IGRA-negative throughout the study and those who showed initial IGRA conversion, but subsequently reverted to IGRA-negative (“IGRA reverters”). In the “reversion-controlled analysis” sustained IGRA converters will be compared to IGRA reverters only. The all mITT-controlled analysis includes participants who remained IGRA negative throughout the study, who likely include a combination of non-exposed individuals as well as exposed individuals in whom vaccine-induced responses or natural immunity were able to prevent initial infection. The latter group is impossible to unequivocally identify in this study since exposure (e.g., to household members with active disease) was not measured for participants. The reversion-controlled analysis on the other hand includes subjects who were clearly exposed since they showed initial IGRA conversion, but then presumably were able to control infection, allowing them to revert to IGRA negative. This analysis therefore controls for exposure, although it does exclude exposed participants who do not convert. In both types of analysis, immune markers that are enriched in vaccine recipients who do not become sustained converters may be identified as putative CoPs. The total number of participants in each of the groups is shown in Figure 3 (n=57 cases and n=187 non-cases).

For the M72/AS01_E_ Program, all cases (n=39) meeting the primary TB disease endpoint definition will be included [20]. A higher non-case to case ratio of samples has been proposed for the M72 study (5:1, n=195 non-cases) compared to the BCG study (3:1) to increase statistical power [57] and adapt for the fewer number of cases in the M72 study (Table 1). Since the BCG study was conducted at two clinical sites in the same province in South Africa [58], no site-specific considerations were required for the selection of non-cases. However, the M72/AS01_E_ trial was conducted at 11 clinical sites across three African countries and significant differences in immunogenicity were noted when comparing participants recruited in South Africa and Kenya [20]. Recruitment site will therefore be an important variable to consider in the selection of non-cases.

CoPs will be evaluated using a range of statistical methods including covariate-adjusted regression to evaluate correlates of risk within each treatment group, a vaccine efficacy modification or “principal stratification” framework to estimate vaccine efficacy as a function of the vaccine-induced biomarker [56, 59], a causal mediation framework to estimate the proportion of the efficacy that can be explained by the biomarker [60] and a machine learning framework, which frames the analysis as a multivariate classification of cases vs. non-cases [61]. Analyses will leverage existing code that was developed and implemented for studying CoRs and CoPs in the US government COVID-19 vaccine trials, including an open-source statistical analysis plan and open-source codebase [62]. Similarly, all Primary analyses will be pre-specified in a statistical analysis plan [63].

Exploratory analyses will also be conducted to evaluate a broader set of potential biomarkers and to infer mechanistic insights from their attributions to protective immunity. With multi-scale cross-cell type “omics” data generated in the case-cohort studies, an integrative multivariate framework that directly models data from several platforms will provide an insightful view of the cross-talk between immune cell types while simultaneously identifying new candidate biomarkers. Specifically, supervised integration models will identify a set of biomarkers that maximize the covariance with phenotypic outcomes while considering interactions among multiple data modalities. In addition, a module-based method transforming genes/proteins/metabolites into pathways/cellular responses based on prior biological knowledge will be integrated into the multivariate modeling to improve data interpretation. As there are many new emerging multi-omic predictive algorithms [64, 65], we are benchmarking existing algorithms holding robust performance and applicable to multi-scale cross-cell type data modalities. Moreover, network analyses through partial correlation networks or Gaussian/mixed graphical models from data integration will also be performed to infer potential functional linkages between immune responses and vaccine efficacy. These descriptive analyses will generate novel hypotheses that may be evaluated in future studies.

## Progress and hurdles

The BCG Program was launched in late 2018, pilot studies have been completed, data review is expected in October 2022, followed by a swift selection of the assays that will be included for the Primary analysis, which should be completed in 2023. The M72/AS01_E_ CoPs Program was launched in early 2020 and Pilot studies are expected to start in late 2022. Both TB Immune Correlates Programs have been heavily affected by disruptions caused by the COVID-19 pandemic, including closure of laboratories in 2020 and general institutional de-prioritization of non-COVID-related research. Additionally, recent changes in South African data privacy legislation (the Protection of Personal Information Act [POPI Act], https://popia.co.za/) resulted in significant delays in the fulfilment of regulatory and contractual requirements due to the unfamiliarity of participating research institutions with the requirements set forth in the act.

The decision to issue open calls for proposals and to establish two large consortia of international investigators was made to ensure that the best possible scientific expertise and state-of-the-art technologies were deployed in the fight against TB, with a spirit of collaboration and data sharing. The hurdles in fulfilling regulatory and contractual requirements involving multiple partners in a timely fashion were initially under-estimated and resulted in significant delays.

## Concluding remarks

The year 2018 was dubbed “the year of TB vaccines” reflecting the publication of vaccine efficacy results for BCG revaccination (POSI) and M72/AS01_E_ (POD). The scientific community has been waiting in hopeful anticipation for the identification of immune correlates of protection against TB, which can be discovered now that randomized placebo-controlled clinical trials of partially efficacious vaccines have been completed. The COVID-19 pandemic has greatly affected TB patient management and research programs around the world, and caused much frustration within the TB research community, which has been chronically under-funded and de-prioritized, despite focusing on the infectious disease that has killed most humans (and continues to do so) in history. The speed and success of COVID-19 research is inspiring, and provides new hope that when vaccine developers, funders, and scientists establish collaborative partnerships with strong political and public support the unimaginable (several COVID-19 vaccines developed at warp speed and identification of the first correlates of protection months thereafter) can happen [62, 66]. This sense of urgency and scale now needs to be applied to TB vaccines.

## Translation Insight

A CoPs for one or both of these TB vaccines has enormous potential to accelerate vaccine development and the clinical development pathway. A “mechanistic CoPs” [67], established by these studies and validated in follow-up experiments, can inform the field about the roles of host immune responses in *Mtb* infection and disease progression, thereby facilitating iterative vaccine design and prioritization of vaccines in the clinical pipeline. However, a CoPs does not need to be mechanistic to be clinically valuable; a validated statistical CoPs - with known or unknown protective mechanisms - can be used to de-risk clinical development and accelerate vaccine licensure through “immuno-bridging”. With a validated CoPs a vaccine could even be licensed on the basis of meeting specific immunogenicity criteria developed from CoPs studies of a previously licensed vaccine [68]. Therefore, establishing a CoPs for PO(S)I or POD based on the BCG and M72 studies could have implications for TB vaccine development well beyond these two vaccines. Candidate CoPs identified by the studies described here will require independent validation, which is possible by leveraging a larger BCG re-vaccination POSI trial ongoing in South Africa (NCT04152161) and a phase 3 trial with M72/AS01_E_, which is currently being planned.

## Figures and Tables

**Table 1:** Summary of BCG revaccination and M72/AS01_E_ vaccine trials

	BCG revaccination POSI trial	M72/AS01_E_ POD trial
Intervention	BCG vaccine, 1 intradermal injection of 5×10^5^ CFU at Day 0	M72/AS01_E_, 2 intra-muscular injections of 10μg M72
Formulation	Live attenuated *M. bovis* (Danish strain 1331), reconstituted in Sauton diluent without adjuvant, ~4000 antigens	Subunit vaccine (M72: recombinant fusion protein of Mtb32A and Mtb39A) with adjuvant (Adjuvant System containing MPL, QS-21 and liposome (25 μg MPL and 25 μg QS-21)
Population	659* HIV negative, IGRA negative adolescents, randomized 1:1 to BCG re-vaccination or placebo	3575 HIV negative, IGRA positive adults, randomized 1:1 to M72/AS01_E_ vaccination or placebo
Efficacy	45.4 % (95% CI 6.4 to 68.1)	49.7 (95% CI 2.1 to 74.2)
Case Definition	Sustained IGRA conversion (secondary endpoint)	Culture or PCR-confirmed pulmonary TB without HIV (primary endpoint)
Endpoints	57 total 21/312 in BCG arm 36/310 in placebo arm	39 total 13/1626 in M72/AS01_E_ arm 26/1663 in placebo arm
Vaccine-reactive immune responses in placebo arm	detectable, high variability	virtually undetectable, low variability
Samples for primary CoP analysis: PBMC Plasma Fixed cells from whole blood RNA from whole blood	2 vials at Day 0, 70, Month 6 2 vials at Day 0, 70, Month 6 2 vials at Day 0, 3 (placebo) or 7 (BCG), Month 6 1 vial at Day 0, 3 (placebo) or 7 (BCG), Month 6	6 vials at Day 0, 37, Month 6 2 vials at Day 0, 37, Month 6 2 vials at Day 0, 37, Month 6 1 vial at Day 0, 37, Month 6

Abbreviations: CFU = colony forming unit; MPL = 3-O-desacyl-4-monophosphoryl lipid A; QS-21 = *Quillaja saponaria* Molina, fraction 21

* H4:IC31 recipients not included in CoP analyses

**Table 2:** Experimental approaches used to measure outcomes within each immune compartment

Immune compartment	Outcome measure	Assay
Antigen-specific T-cell responses	Functional, activation and memory profiles	- PBMC-ICS and flow cytometry - CITE-seq and Seq-Well S^3^ or 10x Genomics on sorted T cells*
	Recognized epitopes	- Tetramer staining and flow cytometry* - IFNγ ELISpot*
	TCR repertoire and gene expression	- Single-cell TCR sequencing on sorted cells (SMART-Seq2 or Seq-Well S^3^ or 10x Genomics)* - Immunoseq*
	Secretion of immunomodulatory factors	- Multiplex Protein Detection Assay*
Humoral responses	Ab titers	- Binding Ab multiplex assay (BAMA)
	Ab sub-classes	
	Ab avidity	- Biolayer Interferometry (BLI)
	Fc-mediated functions	- Ab-dependent NK cell activation - Ab-dependent cellular phagocytosis - Ab-dependent complement deposition - Ab-dependent neutrophil phagocytosis - Neutrophil extracellular traps - Ab-depended dendritic cell phagocytosis - Fc receptor binding array
	Ab specificity	- Linear peptide array* - *Mtb* proteome microarrays* - Phage immunoprecipitation*
Donor-unrestricted T cells	Functional, activation and memory profiles	- PBMC-ICS and flow cytometry
	TCR repertoire and gene expression	- Single-cell TCR sequencing on sorted cells (SMART-Seq2 or Seq-Well S^3^ or 10x Genomics)* - Immunoseq* - CITE-seq and Seq-Well S^3^ or 10xGenomics on sorted T cells*
	Phenotype and absolute counts	- Flow cytometry on fixed whole blood cells
Trained innate immunity	Epigenetic profiles	- EpiTOF (mass cytometry) - Single-cell ATAC-seq - Long non-coding RNA qPCR*
	Functional responses	- PBMC-ICS and flow cytometry - CITE-seq and Seq-Well S^3^ or 10x Genomics on bulk stimulated PBMC - Secreted immunomodulatory factors in response to heterologous stimuli (O-Link)
Cooperation between immune compartments	Mycobacterial growth inhibition	- Heterologous macrophage MGIA with autologous plasma - Heterologous whole blood MGIA with autologous plasma - Autologous PBMC and plasma MGIA*
Innate immunity / Milieu	Bulk PBMC functional, activation and memory profiles	- CITE-seq and Seq-Well S^3^ or 10xGenomics* on bulk stimulated PBMC
	Immunophenotype and absolute counts	- Flow cytometry on fixed whole blood cells
	Gene expression profiles and transcriptomic TB signatures	- RNA sequencing on whole blood
	Apolipoproteins and complement	- Targeted LC/MS*
	Lipidomics	- LC-MS/MS*
	Proteomics	- LC-MS/MS*
	Metabolomics	- GC-MS*

Abbreviations: PBMC = peripheral blood mononuclear cells; ICS = intra-cellular cytokine staining; TCR = T cell receptor; Ab = antibody; MGIA = mycobacterial growth inhibition assay; LC/MS = liquid chromatography and mass spectrometry; LC-MS/MS = liquid chromatography-tandem mass spectrometry; GC-MS = gas chromatography and mass spectrometry.

* M72 only
